# Psychophysical profiles in super-recognizers

**DOI:** 10.1038/s41598-021-92549-6

**Published:** 2021-06-23

**Authors:** Jeffrey D. Nador, Matteo Zoia, Matthew V. Pachai, Meike Ramon

**Affiliations:** 1 Department of Psychology, Applied Face Cognition Lab, University of Fribourg, Rue P.-A. de Faucigny 2, 1700 Fribourg, Switzerland; 2 Perceptual Neuroscience Laboratory, York University, Toronto, Canada

**Keywords:** Psychology, Human behaviour

## Abstract

Facial identity matching ability varies widely, ranging from prosopagnosic individuals (who exhibit profound impairments in face cognition/processing) to so-called super-recognizers (SRs), possessing exceptional capacities. Yet, despite the often consequential nature of face matching decisions—such as identity verification in security critical settings—ability assessments tendentially rely on simple performance metrics on a handful of heterogeneously related subprocesses, or in some cases only a single measured subprocess. Unfortunately, methodologies of this ilk leave contributions of stimulus information to observed variations in ability largely un(der)specified. Moreover, they are inadequate for addressing the qualitative or quantitative nature of differences between SRs’ abilities and those of the general population. Here, therefore, we sought to investigate individual differences—among SRs identified using a novel conservative diagnostic framework, and neurotypical controls—by systematically varying retinal availability, bandwidth, and orientation of faces’ spatial frequency content in two face matching experiments. Psychophysical evaluations of these parameters’ contributions to ability reveal that SRs more consistently exploit the same spatial frequency information, rather than suggesting qualitatively different profiles between control observers and SRs. These findings stress the importance of optimizing procedures for SR identification, for example by including measures quantifying the *consistency* of individuals’ behavior.

## Introduction

Ability in matching images of unfamiliar face identities varies widely between neurotypical adults^1–5^. At the upper end of this spectrum are super-recognizers (SRs), originally reported by Russell et al.^6^ as possessing exceptional facial identity processing capabilities across a range of sub-processes, including face matching, recognition, and identification. Presently, though, there is limited empirical evidence concerning the factors underlying SRs’ extreme abilities^7,8^.

To date, two aspects ubiquitously present in previous studies have hindered genuine progress in understanding the *mechanisms* underlying SRs’ superior abilities: (i) the use of inconsistent or inappropriate diagnostic criteria for assessing such skills; (ii) absence of studies systematically varying the information conveyed by face stimuli themselves. Here, with the goal of addressing these aspects, we studied a sample of SRs drawn from a large cohort of 70 individuals identified with a recently proposed conservative “diagnostic framework” for SR identification^8^. Two experiments systematically varied the spatial frequency content of face stimuli (in terms of its retinal availability and orientation), thus providing a greater level of granularity than previous studies. Moreover, they provide sufficient granularity at the group level to investigate whether previously reported performance differences between SRs and control observers are of a qualitative or quantitative nature. Operationally, this can be framed as asking whether, relative to neurotypical control observers, SRs exploit the *same type of information more efficiently*, or utilize *markedly different types of information*.

### Determining superior face processing abilities

In previous work, identification of individuals as SRs has involved application of simple performance criteria on laboratory-based tests of face identity processing (which we refer to as face cognition or processing in the present work). As mentioned, Russell et al.’s^6^ seminal study reported a small number of SRs who achieved superior performance on three tests of face cognition measuring perceptual matching, old/new recognition and identification. Naturally, such extreme cases are, per definitionem, a relative rarity; so, more thorough multifactorial examination of their performance was critical to the success of their approach. In particular, identifying such individuals among the general population would require scrutiny of the factors that reliably distinguish them.

Yet, in the intervening time, departing from this multi-test procedure, the majority of studies since Russell et al.^6^ have adopted less stringent criteria. Assessments of ability that qualify observers as SRs have for the most part hinged on only a single test and/or process measured: Some have identified SRs based on performance surpassing two standard deviations above the mean of “typical” controls^9–11^ on the long (challenging) version of the Cambridge Face Memory Test (CFMT+; Russell et al.^6^). Others identified SRs based on a high score on “any [test reported] in the super-recognizer literature”^12^.

Such a single-criterion approach to SR diagnosis is problematic for at least two reasons. First, some tests that have been used as a criterion for SR identification are either inappropriate, or often insufficiently sensitive due to relatively low task difficulty. This applies to the short (72-item) version of the CFMT, which was developed for the diagnosis of developmental prosopagnosia^13^, and extends to the CFPT^14^ and GFMT^15^—tests for which individuals with developmental prosopagnosia can even achieve normal performance accuracy^6,16,17^. Tests intended to be sensitive to individual performance differences at the lower end of the performance spectrum lack sensitivity to individual differences at the upper end of the performance spectrum and vice versa, owing to ceiling and floor effects, respectively^17^. Since even the originators of the CFMT+ and CFPT conclude that SRs “are about as good (at face recognition) as many developmental prosopagnosics are bad” (p. 256), it is important that any criteria for SR assessment reflect individual differences at least as large at the upper end of the performance spectrum. Given that the above tests cannot do so alone, multidimensional assessments would be theoretically better suited to this end^8^.

Second, it is well-established that at the individual level, superior performance on one face cognition task/subprocess generalizes poorly, if at all, to others^4,5,9,18–20^. Consequently, performance on any *single* task/sub-process should not be considered as sufficient evidence of processing superiority to warrant generalizing *across* tasks/sub-processes. Therefore, minimally, multiple measures should be applied for SR identification, to ensure that any evidence of superior performance within an individual is *consistent* for various tasks/subprocesses, and across the most challenging of available tests. That is, if SRs are considered to display superior face processing abilities, generally, then their assessment should ideally also encompass their consistency relative to typical control observers for any such task.

Of note, practical concerns potentially used to justify simplified and varied diagnostic assessments (e.g., time constraints, desire to recruit larger SR samples, etc.) would inherently trade off against replicability. Heterogeneous criteria between studies produce concomitantly heterogeneous data. Meanwhile, relatively lax criteria and/or insufficiently difficult tests would lead to increased rates of individuals incorrectly identified as SRs (i.e., false positives), which would in turn contribute to the heterogeneity reported in the SR literature^7,8^. Therefore, reduction of false positives—and by extension the advancement of our understanding of SRs’ abilities—requires a more conservative and widely agreed upon framework for their diagnosis^7,8,21^. Moreover, these criteria should be evaluated within the context of cross-task consistency *within* observers, for a variety of tasks/processes^4,5,7^, in order to determine whether SRs indeed live up to their namesake in distinguishing themselves from the general population.

In the sense that diagnoses are thought to reflect identification based on agreed upon criteria with high sensitivity, which are lacking across studies of SRs, some researchers may deem it premature for any publication to report diagnosis of SRs. Others may consider the term “diagnosis” appropriate exclusively in seeking to identify the presence of an impairment or disease. Of note, numerous studies “diagnose” developmental prosopagnosia, despite the still lacking consensus regarding diagnostic criteria. Additionally, even criteria that have once been generally accepted change historically, while the diagnoses as such prevail. Therefore, acknowledging its origin, we conceptualize “diagnosis” as the systematic investigation conducted with the aim of increasing knowledge by distinction and delineation from other possible phenomena or manifestations. Previous work (including our own) has thus collectively attempted to assess performance for the sake of *identifying* or *diagnosing* SRs.

### Psychophysical descriptions of observers’ information exploitation

Diagnostic issues aside, previous empirical studies of SRs would generally benefit from addressing the two issues raised earlier: a general lack of systematic variations of any parameters across the full-face stimulus, and consideration of only simple performance measures rather than stimulus information-dependent performance profiles. Consequently, these studies are left hard-pressed to answer the currently open question of whether control observers and SRs differ qualitatively, i.e. due to exploitation of distinct types of information^22–24^, or quantitatively, e.g. due to differential exploitation of the same local, featural information available to control observers^25^.

**Figure 1:**
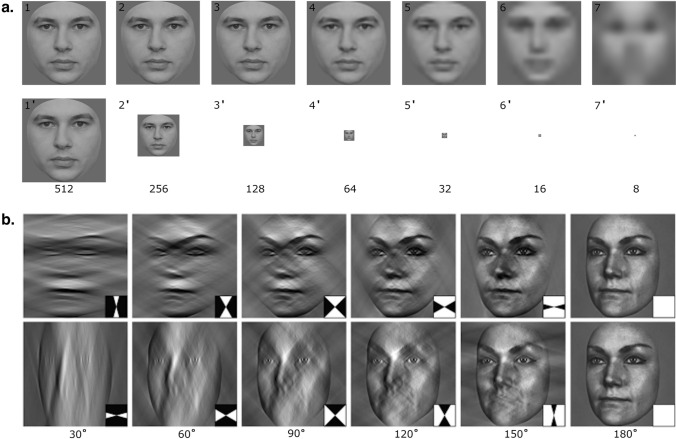
Examples of stimuli used in Experiments 1 and 2. (**a**) In Experiment 1 image size varied logarithmically from 512 pixels in width/height (images 1 and 1′) to 8 pixels (images 7 and 7′). The top row of a. shows the effect of the laplacian pyramid on SF con tent, and the bottom row displays the actual stimulus size. (**b**) In Experiment 2, images were bandpass filtered to preserve horizontal (top row of (**b**)) or vertical (bottom row of (**b**)) information in 15° steps from 0° to 180° (with every second step shown here). (**b**) was reproduced from Pachai et al.^31^.

In general, observers’ performance on tasks employing systematic variations of stimulus parameters inherently derives from the information content they can exploit^26^. For instance, increased viewing distance shifts the bandwidth of spatial frequency information conveyed to the retina^27,28^ (Fig. 1a), until it falls beyond the retina’s contrast sensitivity, at which point even personally familiar face identification performance deteriorates to chance^29^. Crucially, as the spatial frequency content shifts in this way, the identity information (or signal) it conveys is gradually lost following a psychometric function. Psychophysically, the maximum slope of the psychometric function directly relates to the standard deviation of the probability density function: the greater the cumulative distribution’s slope, the less variability is present in its corresponding probability distribution^30^. Operationally, those with steeper psychometric slopes more consistently exploit and utilise the available stimulus information in their judgments. Thus, psychophysical assessment of SRs provides a potentially useful window into the *consistency* with which they exploit stimulus information relative to others, beyond their simple ability (typically measured by the proportion of correct responses alone).

Tardif et al.^25^ recently provided the first experimental psychophysical evidence of differential spatial frequency information exploitation in face stimuli between SRs and control observers. They reported that SRs’ superior performance was related to exploitation of the same local facial feature information as control observers^25^. However, this could also be explained by more *consistent* (i.e., less variable) information exploitation among SRs. Moreover, due to the ‘Bubbles’ response classification method^23^ used by Tardif et al.^25^, (which involves provision of only piecemeal local featural information) usage of spatial frequency information as would be available to the retina under normal viewing conditions^28^ was not probed. Previous studies with typical observers have shown familiarity-dependent enhancement of selectivity for retinally available horizontal, but not vertical, spatial frequency information across the whole face stimulus (Fig. 1b) for upright identity matching^31^. However, this effect has never been assessed in SRs.

In Tardif et al.’^25^ study, both groups of observers had equal access to the information preserved after applying Bubbles filters to stimuli, and were thus limited to use the same information content in making their judgments on each trial. Since SRs generated more hits under the same information constraints, they had to have exploited the same information that controls did, but more effectively. The authors reported that SRs’ performance could be predicted from the same information as controls’, without loss of generality in their model. This further suggests that the difference between the two groups was purely quantitative—SRs exploited the same information as controls while generating more hits. Since adding the same information produced greater gains in performance among SRs, this implies that they would have had steeper psychometric slopes (had those been measured), and would have meant that they were more consistent, according to our definition.

### Considering consistency

Therefore, the present study aims to psychometrically assess individual differences in the *consistency* of full-face spatial frequency and orientation information exploitation, across two experiments. In the first, we test whether consistent exploitation of retinally available spatial frequency information (across simulated viewing distances)^27^ during unfamiliar face matching differentiates SRs from a sample of control observers. In the second, testing the same two groups of observers, we assess whether such (potential) differences extend to increased consistency in horizontal versus vertical spatial frequency structure as shown in previous research^31–35^.

Acknowledging the expressed need to “investigate systematic, functional relationships as they are manifested at the individual participant level…us[ing] methods that are optimized to identify relationships of this kind”^36^ we provide a systematic description of SRs as a unique group of observers. We sought to determine whether these SRs, previously identified using novel conservative criteria^8^, are more sensitive to retinally available spatial frequency information across orientations, as well as whether they more consistently use information diagnostic for facial identity, exploiting it to enhance their judgments of facial identity.

## Method

All procedures and protocols were approved by the University of Fribourg’s Ethics Committee (approval number 473) and conducted in accordance with both their guidelines, as well as those set forth in the Declaration of Helsinki. All participants were healthy volunteers, provided informed written consent and were not financially compensated for their participation.

### Participants

Thirty-one students in the Department of Psychology and the University of Fribourg (21 Females; mean age of 27 years; age range of 20–47 years) participated in two experimental sessions in exchange for course credit. SRs who participated in this study were recruited from a larger cohort of recently reported super-recognizer individuals^8^. In short, they were identified as disposing of exceptional face processing ability if they achieved high performance (relative to previously tested normative samples)^4,5^ on at least two of three challenging tests of face cognition: the YBT (long form)^4,5,37^, the FICST^4,5,38^, and the CFMT+^6^. As previous work has shown that high performance on one test does not predict high performance on another test^4,5^, the requirement of surpassing typical performance on at least two of the three extremely challenging tests represents a more conservative diagnostic approach relative to most previous selection methods^8^. No participants in either group were personally familiar with any of the identities of the face stimuli presented throughout the experimental sessions, and all were eye disease-free, with normal or corrected-to-normal visual acuity, as assessed by qCSF^39,40^ (see Supplemental Material, Table S1).

### Apparatus

Psychophysical experiments were designed and implemented using the PsychToolbox^41–43^ in the Matlab (Mathworks Inc.) programming environment. Two different monitors were beta calibrated and employed for stimulus presentation throughout experimental sessions: a VIEWPixx/3D—22005C monitor (1980 × 1020 pixel resolution; viewing distance: 68.5 cm; 120 Hz refresh rate; average luminance of ca. 110 cd/m^2^), and a Samsung SyncMaster 2233RZ—3D LCD monitor (1680 × 1050 pixel resolution; viewing distance: 62.2 cm; 60 Hz refresh rate; average luminance of ca. 110 cd/m^2^)^44^.

### Procedures

In both experiments, participants were instructed to complete a 10AFC sequential face matching task as employed previously^31^. On each trial throughout the task, they had to match one initially presented left- or right-facing filtered target identity to one of 10 unfiltered front-facing potential probe identities. The beginning of each trial was signaled by the appearance of a central fixation point (1 s duration), whose disappearance (for 100 ms) in turn signaled the appearance of the target identity (250 ms duration). This sequence was immediately followed by the aforementioned array of 10 potential probe identities, in two horizontal rows of 5 (with males above and females below fixation). Participants were given unlimited time to select one probe that (best) matched the target’s identity, by hovering the mouse cursor over it and pressing the left mouse button to confirm their selection. They were further instructed to prioritize responding correctly over quickly. A matching identity was always presented among the 10 potential probes (i.e., the experiment did not include target-absent trials), while four other same-sex and five opposite-sex identities were randomly drawn from the remaining face identities in the stimulus set to make up the array of potential probes. A fully counterbalanced and randomized sequence of images was presented to each participant. Each session of Experiment 1 consisted of 480 trials, lasting roughly 45 min total, including five mandatory break intervals (of participant-determined duration) to alleviate fatigue. All procedures in Experiment 2 were identical, with the exception that the fully counterbalanced and randomized sequence of stimuli comprised 350 trials and 4 break intervals.

### Stimuli

#### Experiment 1

Twenty-five male and 25 female faces, sampled from a larger database of images taken from students at Université catholique de Louvain (Belgium) who provided written consent for their image usage, served as the experimental stimulus set^45^. For each of these face models, three images were taken from different viewpoints, then converted to grayscale and cropped to exclude external identifying features (e.g. ears, hair) (Adobe Photoshop, Adobe Inc.), centered in a 512 × 512 pixels array delimiting all stimuli to 5.4° of visual angle. One front-facing, one left-rotated and one right-rotated viewpoint were generated per face identity, yielding 60 unique images. Each was then isotropically reduced in scale to each of 7 different pixel sizes (8, 16, 32, 64 and 512 pixels across) as a manipulation of viewing distance, using the Laplacian pyramid model^27,28^. This simulates the retinally available information at each size-correspondent viewing distance by effectively removing high spatial frequency content at larger viewing distances (see Fig. 1a). All stimuli were finally adjusted to a root-mean-squared contrast of 0.2.

#### Experiment 2

Participants were shown the same 10 upright male and 10 upright female identities as used previously^31^, including 3 different viewpoint images of each identity, as in Experiment 1. Here, however, rather than reducing the images’ scale as in Experiment 1, we systematically varied the amount of horizontal and vertical information present in these images. All were band-passed using sharp-edged orientation filters centered on 0° (for horizontal information filtering) or 90° (for vertical information filtering) (Fig. 1b). Then, all were filtered at each of 12 bandwidths in steps of 15° (15°, 30°, 45°, 60°, 75°, 90°, 105°, 120°, 135°, 150°, 165°, 180°), such that 180° filters passed all horizontal and vertical information (unfiltered stimuli), while 90° was the largest bandwidth at which horizontal and vertical information was independently isolated^31^.

### Logistic regression analysis

In order to assess individual and group level differences in psychophysical performance, we employed mixed effects (fixed and random) logistic regression models using Matlab’s (Mathworks) nlmefit function. These were constructed incorporating the experimental parameters (e.g. bandwidth, image size) of interest, separately for each experiment, as predictors of correct responses. As a first step, we selected parameters for the base model (e.g. not accounting for group) based on minimization of the Akaike Information Criteria (AIC) that evaluate model parsimony (quality of fit, given the number of parameters required to achieve it). We subsequently compared the Bayesian Information Criteria (BIC) between optimally parameterized models hierarchically, as an estimate of the Bayes factor, in order to determine the effect of each predictor’s inclusion on the relative goodness of fit of the competing models. In general, this means that we evaluated each predictor’s effect by fitting one model that included it, and compared it with the model excluding it, but that was otherwise identical. Each model fitted a logistic function to the averaged data as an approximation of the psychometric function, and each parameter φ_ijk_ could include a fixed effect for the jth group as well as a random effect for the ith observer:

1 $$ \varphi _{{ijk}}  = ~\left( {~\beta _{{jk}} ~ + ~b_{{ijk}} ~} \right)~x_{{ij,}} $$

where k = 1 corresponded to the scaling factor, k = 2 to the mean, and k = 3 to the slope of the psychometric function.

In order to assess contributions of the latent grouping variable, each parameter was subdivided to take on nonzero values only when fitting the *i*th *j*th group by letting φ_*i1k*_ = 1 for SRs, and 0 otherwise, and similarly letting φ_*i2k*_ = 1 for control observers and 0 otherwise. Thus, the full model’s logistic regression function had the general form:

2 $$ \user2{~}\hat{y} = \frac{{\varphi _{{ij1}} }}{{~e^{{\left( {x - \varphi _{{ij2}} } \right)/\varphi _{{ij3}} }} }}~~ + ~\left( {1 - \varphi _{{ij1}} } \right)~ + ~\varepsilon , $$

Similar parameterization was employed for filter orientation in Experiment 2 (though at the subject level, rather than group level).

## Results

### Experiment 1

In order to assess whether SRs exploit available SF information differently from control observers, as well as to generate psychometric functional fits to individual observers’ data (see Supplemental Material, Fig. S1, left panels for model fits to individual observers’ data), we fitted three mixed effects logistic regression models to the trial-level data (see Table 1 for descriptions of models and their comparative fits to the data). Here, image size and group were taken as predictors of correct responses. In general, we evaluated each effect by fitting one model including it, and compared it with a model excluding it, but that was otherwise identical.

**Table 1:** Model comparison—retinally available SF information.

	Parameters	Predictors	Model 1		Model 2	
			ΔAIC	BF k	ΔAIC	BF k
Model 1	Scale	Image size				
Model 2	Slope, scale	Image size	− 913.9	197.7	–	–
Model 3	Slope, scale	Image size, group	–	–	− 87	1.2

Each cell describes model comparisons according to row and column. The BF k index is calculated as log10 BF, such that positive values of BF k reflect evidence in favor of the row model, and negative k values reflect evidence in favor of the column model. ΔAIC scores reflect changes in model parsimony (a function of the fit, given the number of parameters added in the row model relative to the column model). In general, negative values reflect more parsimonious fits for the row than column model and vice-versa.

The first model included regression coefficients for fixed and random effects at the subject level, though not the group level, and thus assessed the effect of retinally available SF information on performance at the subject level, irrespective of group. Including both a slope parameter and a scaling parameter (Model 2) resulted in a more parsimonious model fit than including only a scaling parameter (Model 1) (ΔAIC_21_ =  − 913.9), suggesting that individual differences in performance were best accounted for by changes in slope (BF_21_ = 2.49 × 10^23^; BF k > 10). The third model therefore included parameters for the fixed and random effects of group, for both slope and scale. Model comparison revealed that inclusion of group-level effects in Model 3 more accurately predicted performance than model 2 (BF_32_ = 739.5), which was blind to group effects.

Taken together, these results suggest that SRs’ performance differs from controls. To determine whether there were any consistent differences in slope between groups, we performed an independent-samples post-hoc t-test on the slope parameters obtained for both groups, and found that SRs had significantly steeper psychometric slopes than controls (t(40) = 2.10, p = 0.04).

Overall, the fact that models tended to reduce the AIC when adding parameters suggests that we were not ‘overfitting’ the data with their addition. That is, the addition of parameters tended to produce better fits, and not at the expense of providing parsimonious explanations of the data.

### Experiment 2

In order to assess whether psychophysical performance (the probability of correctly responding as a function of filter bandwidth) varied as a function of slope or scaling factor, we fitted two mixed effects logistic regression models to observers’ trial-level data, as in Experiment 1 (see Table 2 for descriptions of models and their comparative fits to the data). Model 2, accounting for both slope and scaling factor, predicted correct responding more parsimoniously than Model 1 (ΔAIC_21_ =  − 7.4, BF_21_ = 7.0), which accounted only for the scaling factor. We therefore retained predictors for slope and scaling factors in subsequent models assessing horizontal selectivity and group effects.

**Table 2:** Model comparison—orientation and bandwidth of spatial frequency information.

	Parameters	Predictors	Model 1		Model 2		Model 3		Model 4	
			ΔAIC	BF k	ΔAIC	BF k	ΔAIC	BF k	ΔAIC	BF k
Model 1	Scale	Bandwidth								
Model 2	Slope, scale	Bandwidth	− 7.4	0.9	–	–	–	–	–	–
Model 3	Slope, scale	Bandwidth, orientation	–	–	− 631.5	135	–	–	–	–
Model 4	Slope, scale	Bandwidth, group	–	–	− 2.1	1.8	629.5	− 136.7	–	–
Model 5	Slope, scale	Bandwidth, group, orientation	–	–	629.5	131	− 4.3	− 3.6	− 633.8	133.1

Each cell describes model comparisons according to row and column. The BF k index is calculated as log10 BF, such that positive values of BF k reflect evidence in favor of the row model, and negative k values reflect evidence in favor of the column model. ΔAIC scores reflect changes in model parsimony (a function of the fit, given the number of parameters added in the row model relative to the column model). In general, negative values reflect more parsimonious fits for the row than column model and vice-versa.

**Figure 2:**
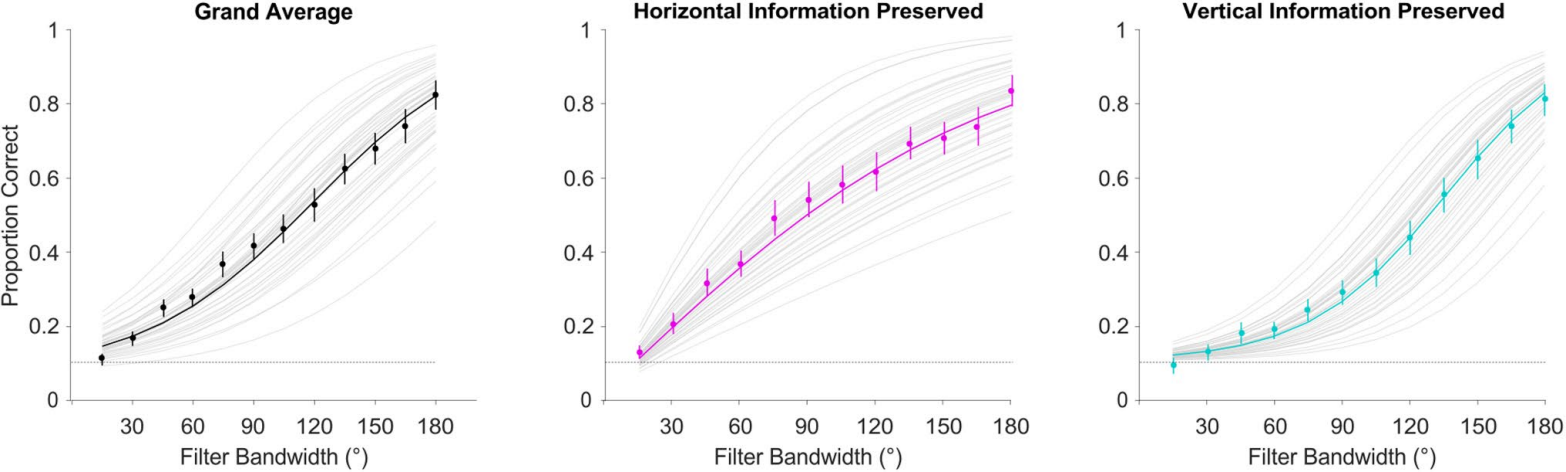
Logistic function fits to trial-level correct responding as a function of filter orientation in Experiment 2. Grand averaged data (left) are plotted alongside Model 2’s fit, with gray tracings displaying individual observers’ fits for comparison. Model 3’s fits to the orientation-split averaged data are plotted separately for images retaining horizontal (middle) and vertical (right) SF information, across filter bandwidth conditions. Error bars represent ± 1SEM; dotted lines represent chance performance.

The third model assessed the effect of horizontal selectivity (Fig. 2, center & right panels), including orientation as a predictor of performance, by comparison against Model 2 (Fig. 2, left panel). These two models were identical except in the respect that Model 3 accounted for filter orientation. Model 3 provided a better fit to the data than model 2 (ΔAIC_32_ =  − 631.5, BF_32_ = 7.39 × 10^134^), suggesting that filter orientation is an important predictor of psychophysical performance, irrespective of group, decisively supporting the effect of horizontal selectivity (see Supplemental Material, Fig. S2 for a visualization more directly replicating previous work^31^).

**Figure 3:**
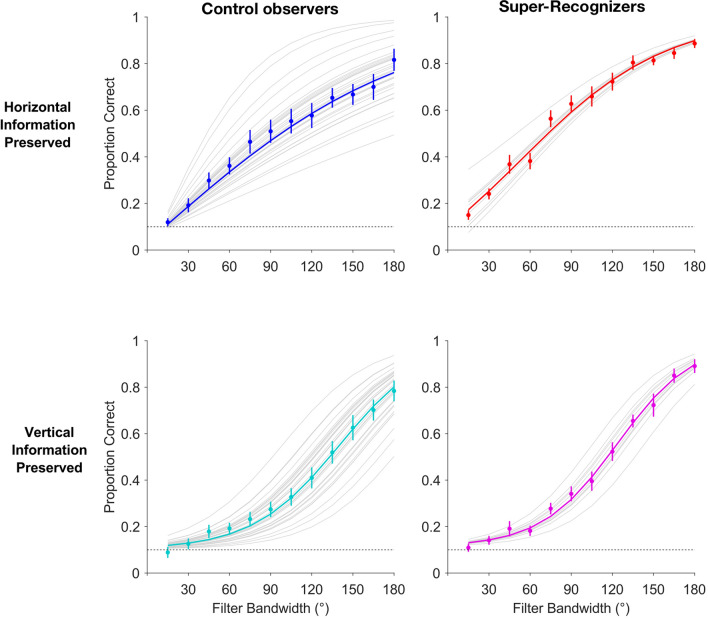
Logistic approximations of the psychometric function fitted to trial-level correct responding, accounting for both Group and Orientation as factors. Averaged data are grouped by column (control observers, left column; SRs, right column), with gray tracings in each displaying individual observers’ model fits for comparison. Averaged data were further split by filter orientation, and are plotted separately for images retaining horizontal (top row) and vertical (bottom row) SF information, across filter bandwidth conditions. Error bars represent ± 1SEM; dotted lines represent chance performance.

Next, we fitted a model (Model 5, c.f. Table 2) that additionally included group-level coefficients in order to distinguish SRs from controls (Fig. 3). The only difference between Models 3 and 5 was the addition of group-level coefficients in Model 5, such that any change in fit between models would have had to arise from between-group differences in horizontal selectivity beyond any variance already explained by the fixed and random effects of horizontal selectivity in the overall sample. Comparison of the models’ fits suggests that Model 3 provided a better overall fit to the data (Fig. 3), decisively suggesting that SRs’ horizontal selectivity is no different from that of control observers (ΔAIC_32_ =  − 3.6, BF_43_ = 2.647 × 10^–4^).

**Figure 4:**
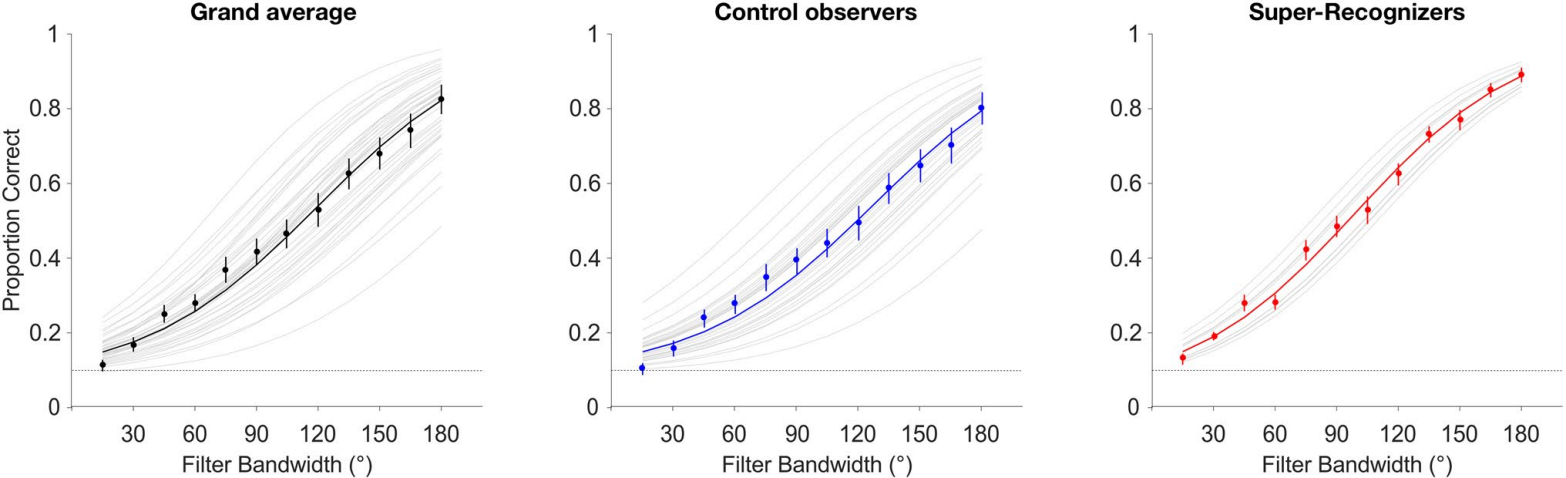
Logistic fits correct responding accounting for Group. Grand averaged data (left) from Model 3 are plotted alongside Model 5’s fits (with gray tracings displaying individual fits to observers’ data) to control observers (middle) and SRs (right) for comparison. Error bars represent ± 1SEM; dotted lines represent chance performance.

Although comparison of models 5 and 3 suggested that SRs did not exhibit differential horizontal selectivity relative to controls, they could show generally increased psychometric slopes relative to control observers, irrespective of orientation. Therefore, we fitted a model that accounted only for group (c.f. Model 4, Table 2), and not for filter orientation (Fig. 4). Comparison of Model 5 (accounting for group and filter bandwidth) against Model 4 (accounting only for bandwidth) revealed that the grouping factor strongly improved the model fit (ΔAIC_65_ =  − 633.8; BF_54_ = 1.3 × 10^133^). A post-hoc t-test of fitted slopes between groups (from model 5) reveals that SRs’ were steeper than control observers’ (t(40) = 3.62, p < 0.0001). Overall, these results suggest that while SRs do not display enhanced horizontal selectivity relative to controls (i.e. evidence favors the null hypothesis that there is no effect of filter orientation), they do tend to exhibit more consistent performance overall (see Supplemental Material, Fig. S1, right panels, for model fits to individual observers’ data).

As was the case in Experiment 1, addition of predictors and parameters to our models tended to reduce the AIC. So, their addition seems not only to produce tighter fits to the data, but to do so without sacrificing parsimony of the model.

## Discussion

In this study we sought to address the currently open question of whether SRs—individuals with exceptional face processing skills^6–8^—exploit spatial frequency information in a quantitatively or qualitatively different manner than typical individuals. To this end, we systematically varied retinal availability, bandwidth, and orientation of faces’ spatial frequency content across two face matching experiments. Together the results reveal that SRs utilize the same information, albeit more consistently than control observers.

The results of Experiment 1 demonstrate that SRs show higher consistency (as evidenced by steeper psychometric slopes) in face matching than controls (Fig. 2), over a similar range of Laplacian Pyramid-simulated viewing distances^27–29^. This suggests that—among the factors examined in the current experiments—SRs more readily incorporate retinally available spatial frequency information in their matching judgments of facial identity. However, absolute identity discrimination thresholds were low enough in both groups of observers to imply that they likely approached the face detection threshold, rendering qualitative differences in the range of spatial frequency information utilization between groups difficult to observe.

Meanwhile, in our second experiment, we find horizontal selectivity commensurate with previous studies^31,32,34,35^, as suggested by improved model fits when adding orientation as a factor (Figs. 2, 3). More specifically, horizontal spatial frequency information is more diagnostic for facial identity matching than vertical, among both groups of observers (Supplemental Material, Figs. S1, S2). SRs’ performance was also quite different from control observers’—across filter bandwidths and orientations—suggesting that they have generally higher sensitivity to spatial frequency content when matching facial identity. This is indicated by the group-level differences in psychometric slopes, irrespective of orientation (Fig. 2). And critically, in terms of our initial hypotheses, it implies that SRs do not display differential horizontal selectivity from controls. We also find no evidence to suggest that SRs employ qualitatively different spatial frequency bands than control observers (see Supplemental Material, Fig. S1 for individual observers’ profiles), though we do not specifically find evidence against this possibility either. So, while we do find evidence of horizontal selectivity as reported by previous research, it is neither particularly predictive of superior ability for face identity matching, nor does it reveal any qualitative differences between groups.

### On quantitative versus qualitative differences in face processing

To our knowledge, the only previous psychophysical study assessing the qualitative versus quantitative nature of SRs’ distinguishing characteristics^25^ used the ‘Bubbles’ response classification method^23,46^. This method applies random spatial frequency bands to random facial locations in a piecemeal fashion, over a large number of trials, reconstructing the classification image based solely on the contribution of un/available local information. These particular stimuli substantially distort the distribution of spatial frequency information available under normal viewing conditions^28^. Despite using manipulations of information applied uniformly across the *full* face, our results nonetheless agree with those previously reported^25^, in that both SRs and typical observers utilized roughly the same range of spatial frequency information. However, here we add explicitly that SRs exploit this information more *consistently*.

**Figure 5:**
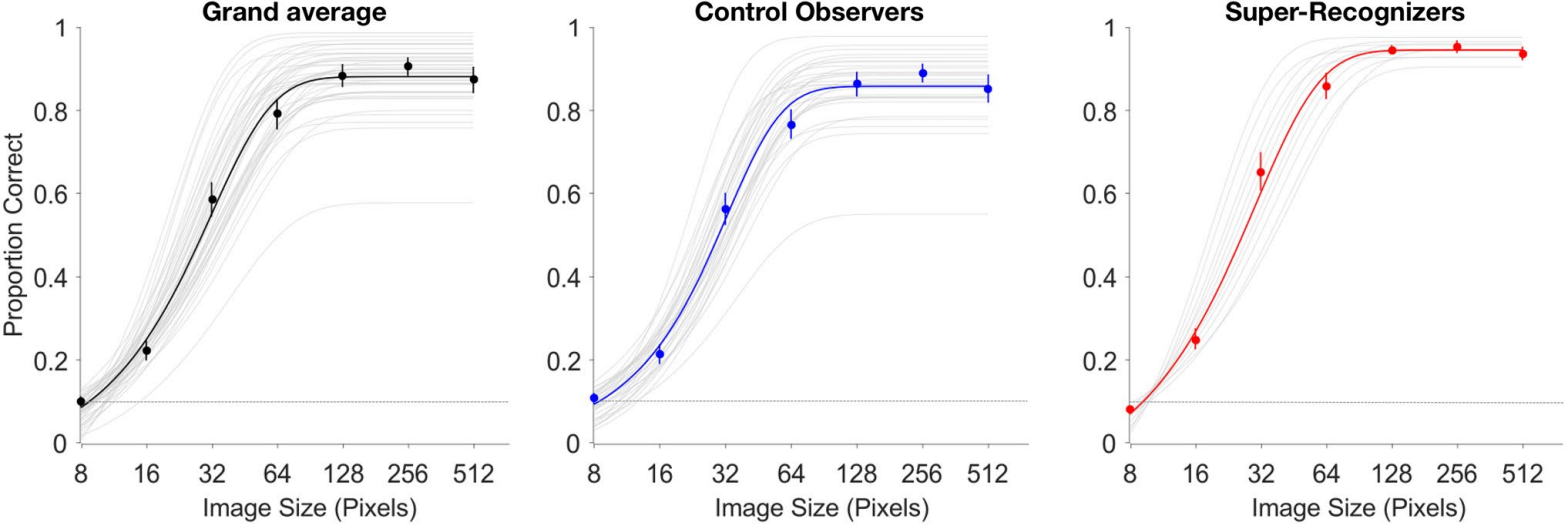
Logistic approximation of the psychometric function fitted to trial-level correct responding for Experiment 1. Grand averaged data (left) are plotted alongside Model 2’s fit, with gray tracings displaying individual observers’ model fits for comparison. Model 3’s fits to the group-averaged data are plotted separately for control observers (middle) and SRs (right) across image size conditions. Error bars represent ± 1SEM; dotted horizontal lines represent chance performance.

This agrees with the individual differences in psychometric functional fits that we find across experiments (c.f. Figs. 3, 5). While both groups (control observers and SRs) show comparable matching thresholds for retinally available spatial frequency content across spatial scales, as well as for horizontally and vertically oriented spatial frequency content, we find that consistency of *retinally available* spatial frequency exploitation (i.e., psychometric slope) predicts SR status (Fig. 5), irrespective of the spatial frequency content’s spatial orientation (Fig. 4).

Overall, we surmise that within-observer consistency constitutes an important aspect of performance, in that it is predictive of superior face processing abilities (Figs. 3, 4, 5), but alone is likely not sufficient for SR identification. The improved model fits upon inclusion of scaling factors generally suggest that some portion of psychophysical performance is not accounted for with slope (and thus consistency) alone. For instance, SRs seem to reach greater asymptotic performance levels than controls, on average, which warrants an investigation into selectivity for information at different spatial scales. Potentially, application of similar psychophysical techniques over a wider array of stimulus properties, spanning the gamut of potential differences in information exploitation between typical observers and SRs, could provide more and clearer diagnostic criteria, and further, possibly reveal qualitative differences unavailable to scrutiny here.

### Consistency as a potentially novel means for super-recognizer classification

Given the paucity of psychophysical studies of SRs’ abilities, we propose that future studies adopt such systematic approaches to provide a more detailed description of their unique skill(s). While we find *consistency* to be predictive of superior ability (as also reported recently in typical cohorts^47^), we would advocate deploying measures of consistency in addition to more traditional performance metrics and not as a substitute for them. That is, they ought to be integrated to extend the granularity of current^8^ multifactorial frameworks of SR diagnostic tests, as originally proposed^6^. Moreover, systematic variations of different kinds of stimulus information hold the potential to reveal other important factors that contribute to overall ability. Recognizing that such procedures are comparatively more cumbersome, we consider their inclusion a crucial step in addressing the stated needs for appropriately stringent diagnostic tools and reduction of false positive SR identification^7,48–50^.

## Conclusion

Over two psychophysical experiments, we tested proficiency for matching of unfamiliar facial identity in a group of 11 conservatively identified SRs and 31 neurotypical observers. Following parametric manipulations of spatial frequency content and orientation across the full face, we find relatively quantitative differences between SRs and control observers. However, it should be emphasized that we cannot rule out the potential for qualitative differences beyond the scope of the parametric stimulus manipulations used here. But, based on the currently available psychophysical findings, SRs should not be described as a special class^51^, but rather as experts among experts. Rather than accepting this notion, we emphasize that further work using complementary methods is needed to ascertain whether SRs process facial information in just quantitatively, or also qualitatively, different ways than typical psychophysical observers. Indeed, it is plausible that qualitative differences—similar to those reported for developmental prosopagnosia^52–54^—may be found in larger cohorts of carefully described SR cases identified using the same multi-factorial diagnostic framework^8^.

## Supplementary Information

Supplementary Information.

## Data Availability

Accompanying data can be downloaded from the Open Science Framework (https://osf.io/5h3rj/).
