# Supplementary material for: Psychophysical profiles in super-recognizers
Source: Sci Rep. 2021 Jun 23;11:13184. doi: 10.1038/s41598-021-92549-6 (PMC8222339; doi:10.1038/s41598-021-92549-6)
Supplement: Supplementary file 1 — Supplementary Information. [file 41598_2021_92549_MOESM1_ESM.pdf]

Supplementary Information for

Psychophysical Profiles in Super-Recognizers

Jeffrey D. Nador, Matteo Zoia, Matthew V. Pachai, & Meike Ramon*

* Correspondence: meike.ramon@gmail.com

Table S1. Demographic information and results of Quick Contrast Sensitivity (qCSF) assessment. Note that data for two control observers and four SRs are missing as we terminated inclusion of qCSF measurement after t-tests between observer groups performed on data from 37 observers revealed no indication of differences for qCSF parameters. The only significant group difference that emerged was for observers' age ($t(42)=8.09$, $p<.01$).

**Observer Demographics**				**Quick Contrast Sensitivity Function (qCSF) Assessment**			
Observer ID	*Gender*	*Handedness*	*Age*	*Peak Sensitivity*	*Peak Frequency*	*Bandwidth*	*CSF Area*
Control Observers							
C1	F	Right	27.00	47.27	5.00	7.35	87.10
C2	M	Right	27.00	39.14	2.26	4.62	22.53
C3	F	Right	21.00	42.21	2.11	6.58	30.84
C4	F	Right	23.00	15.19	1.23	5.54	8.08
C5	F	Right	21.00	17.10	8.46	6.15	65.66
C6	F	Right	21.00	29.13	4.52	7.83	61.61
C7	F	Left	22.00	23.81	3.50	7.20	38.42
C8	F	Right	22.00	34.02	2.52	7.82	37.74
C9	F	Right	20.00	27.50	3.60	3.39	22.23
C10	F	Right	26.00	18.39	6.86	6.66	59.64
C11	F	Right	22.00	28.83	2.60	7.65	34.31
C12	F	Right	20.00	19.40	2.14	4.29	13.15
C13	F	Right	20.00	35.81	2.99	7.11	42.35
C14	F	Right	24.00	8.69	1.01	2.50	2.45
C15	F	Right	27.00	25.52	2.80	8.35	36.65
C16	M	Right	22.00	2.02	2.41	1.14	0.42
C17	F	Right	20.00	33.97	2.38	7.01	32.27
C18	M	Right	24.00	28.98	1.79	4.81	15.75
C19	F	Right	22.00	13.43	7.26	6.69	50.62
C20	F	Left	31.00	22.61	2.06	7.25	21.87
C21	F	Right	23.00	26.94	1.43	6.72	16.07
C22	M	Right	28.00	38.44	3.23	8.67	57.61
C23	F	Right	29.00	29.07	5.72	6.76	68.10
C24	F	Right	23.00	23.35	1.59	5.42	13.42
C25	F	Right	21.00	28.18	2.42	4.52	19.94
C26	F	Right	20.00	20.53	2.06	7.90	22.17
C27	M	Right	30.00	29.51	2.65	8.54	39.10
C28	F	Right	20.00	40.55	1.86	8.83	35.02
C29	F	Right	20.00	28.68	2.68	3.47	17.81
C30	F	Right	21.00	20.50	2.03	5.74	16.50
C31	F	Right	21.00	—	—	—	—
C32	M	Right	30.00	—	—	—	—
		**Control observers' mean**	23.38	26.63	3.11	6.22	32.98
		**Control observers' SD**	3.45	10.08	1.83	1.91	21.16
Super-Recognizers							
MB2	F	Left	45.00	23.66	2.17	8.48	27.25
GP1	M	Right	47.00	29.38	1.40	6.66	16.54
MB1	M	Right	34.00	27.35	2.85	8.09	38.05
PT1	F	Right	33.00	2.10	0.82	1.73	0.24
NC1	F	Right	41.00	29.19	2.83	7.81	38.32
VZ1	M	Right	24.00	37.05	2.46	8.61	42.60
AM1	F	Right	31.00	16.01	2.71	3.13	11.61
FW1	M	Right	32.00	—	—	—	—
MW1	M	Right	43.00	—	—	—	—
UC1	M	Right	42.00	—	—	—	—
CB1	F	Right	32.00	—	—	—	—
		**Super-Recognizers' mean**	36.73	24.51	2.57	6.09	27.31
		**Super-Recognizers' SD**	7.21	11.40	0.78	2.79	15.95

Caption for Supplementary Figure S1a-d. Individual SRs' profiles across experiments. Each observer's performance profile is displayed for Experiment 1 and 2 in their respective left and right panels. For Experiment 2, horizontal and vertical structure are plotted with circles and triangles, respectively. Individual SRs' are referred to using their unique acronyms as provided with their original SR-diagnostic information by Ramon (2021). In each plot, chance performance for 10AFC (0.1 proportion correct responding) is denoted by a dotted line. Error bars represent ±1 SEM for each data point.

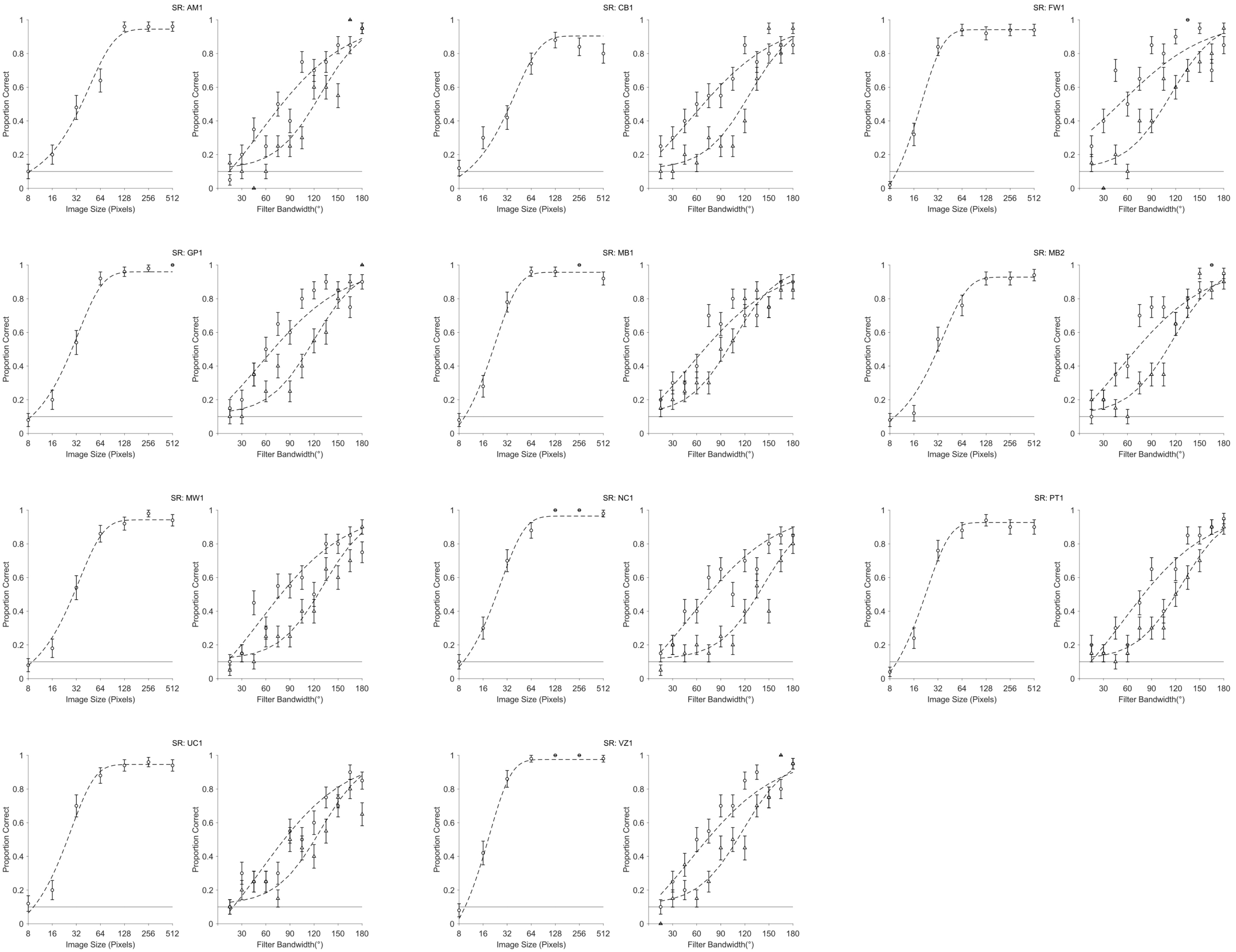
SR: AM1
SR: CB1
SR: FW1
SR: GP1
SR: MB1
SR: MB2
SR: MW1
SR: NC1
SR: PT1
SR: UC1
SR: VZ1
Proportion Correct
0 0.2 0.4 0.6 0.8 1
8 16 32 64 128 256 512
Image Size (Pixels)
30 60 90 120 150 180
Filter Bandwidth(°)

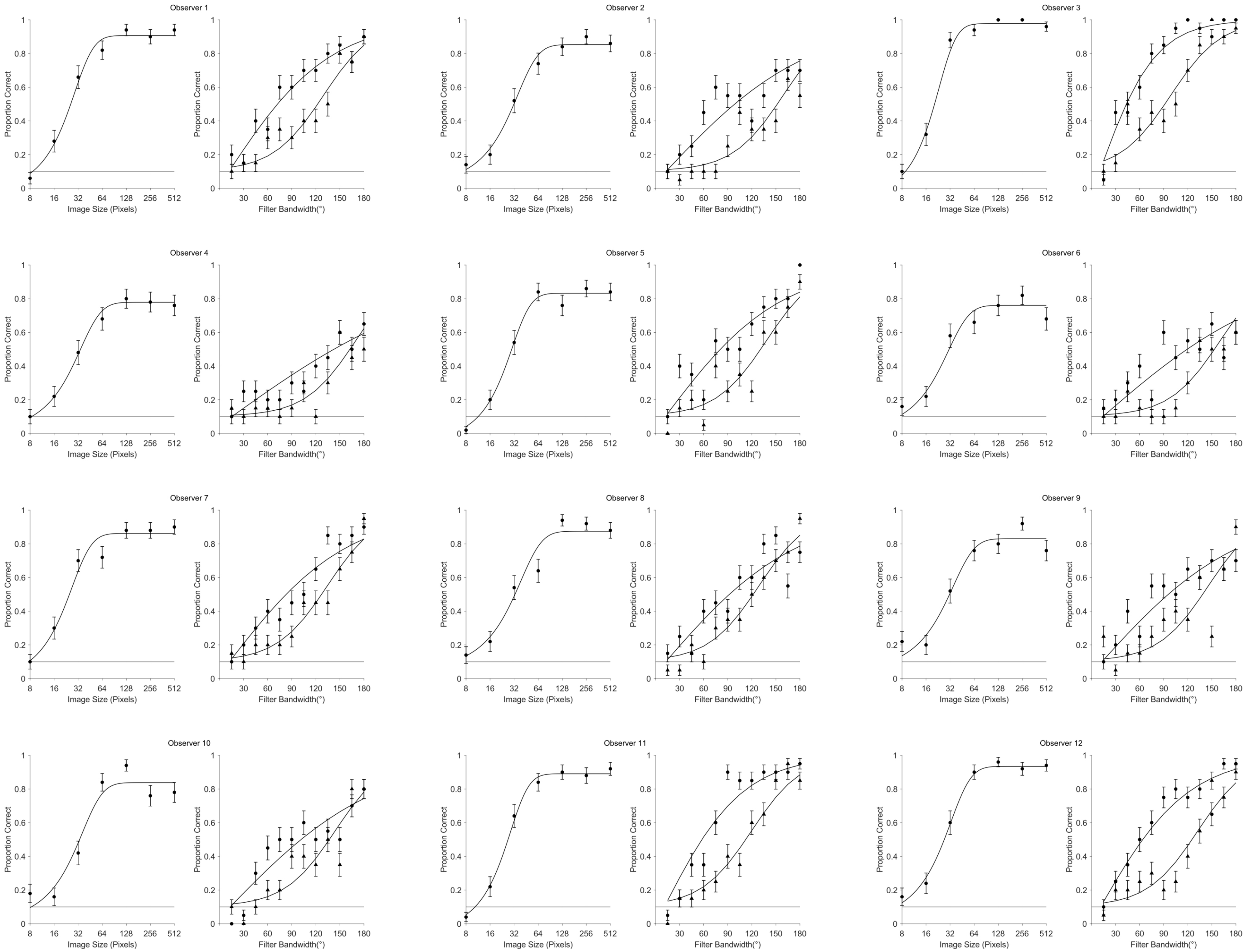

Observer 1
Observer 2
Observer 3
Observer 4
Observer 5
Observer 6
Observer 7
Observer 8
Observer 9
Observer 10
Observer 11
Observer 12
Proportion Correct
0
0.2
0.4
0.6
0.8
1
8
16
32
64
128
256
512
Image Size (Pixels)
30
60
90
120
150
180
Filter Bandwidth(°)

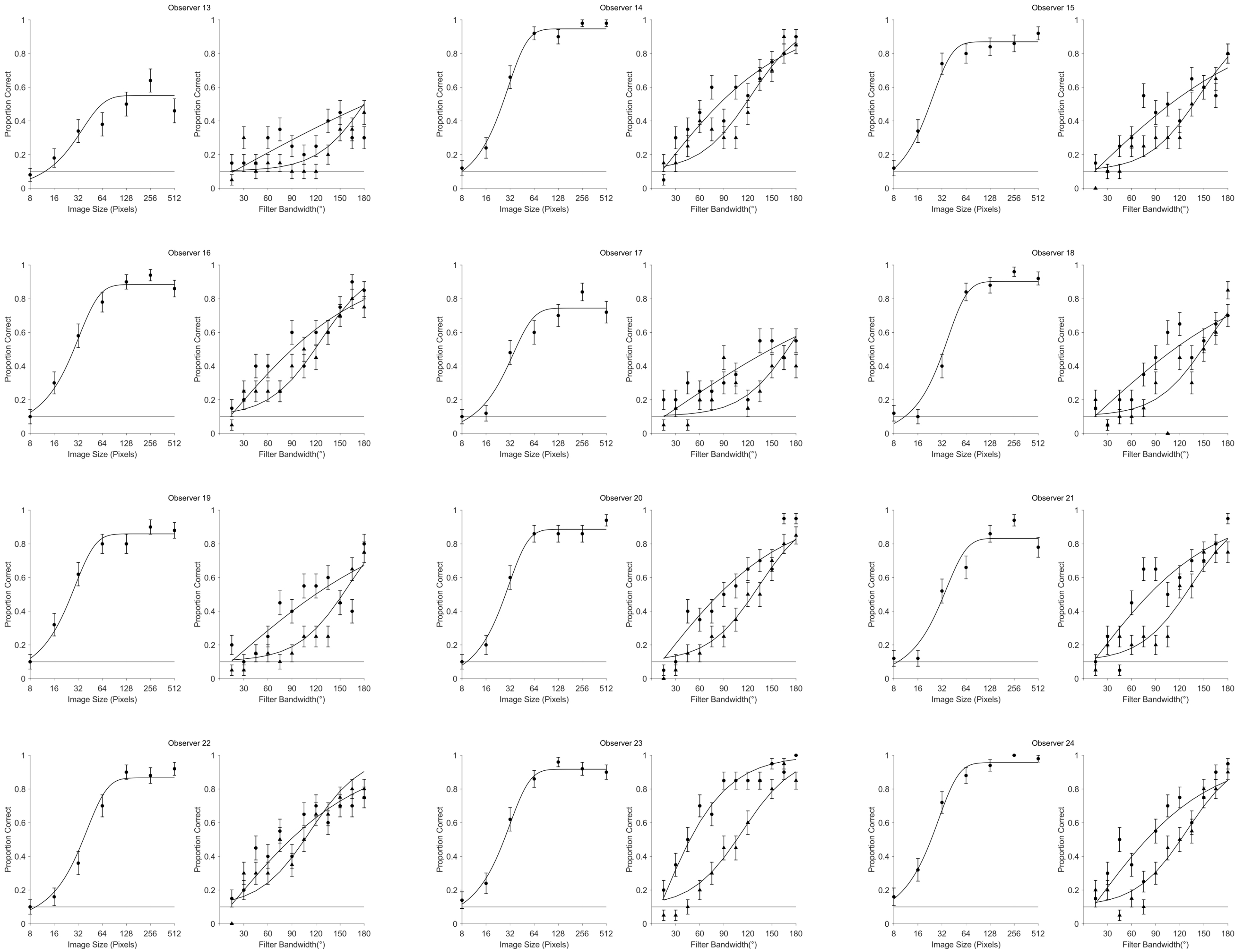
Observer 13
Observer 14
Observer 15
Observer 16
Observer 17
Observer 18
Observer 19
Observer 20
Observer 21
Observer 22
Observer 23
Observer 24
Proportion Correct
0 0.2 0.4 0.6 0.8 1
Image Size (Pixels)
8 16 32 64 128 256 512
Filter Bandwidth(°)
30 60 90 120 150 180

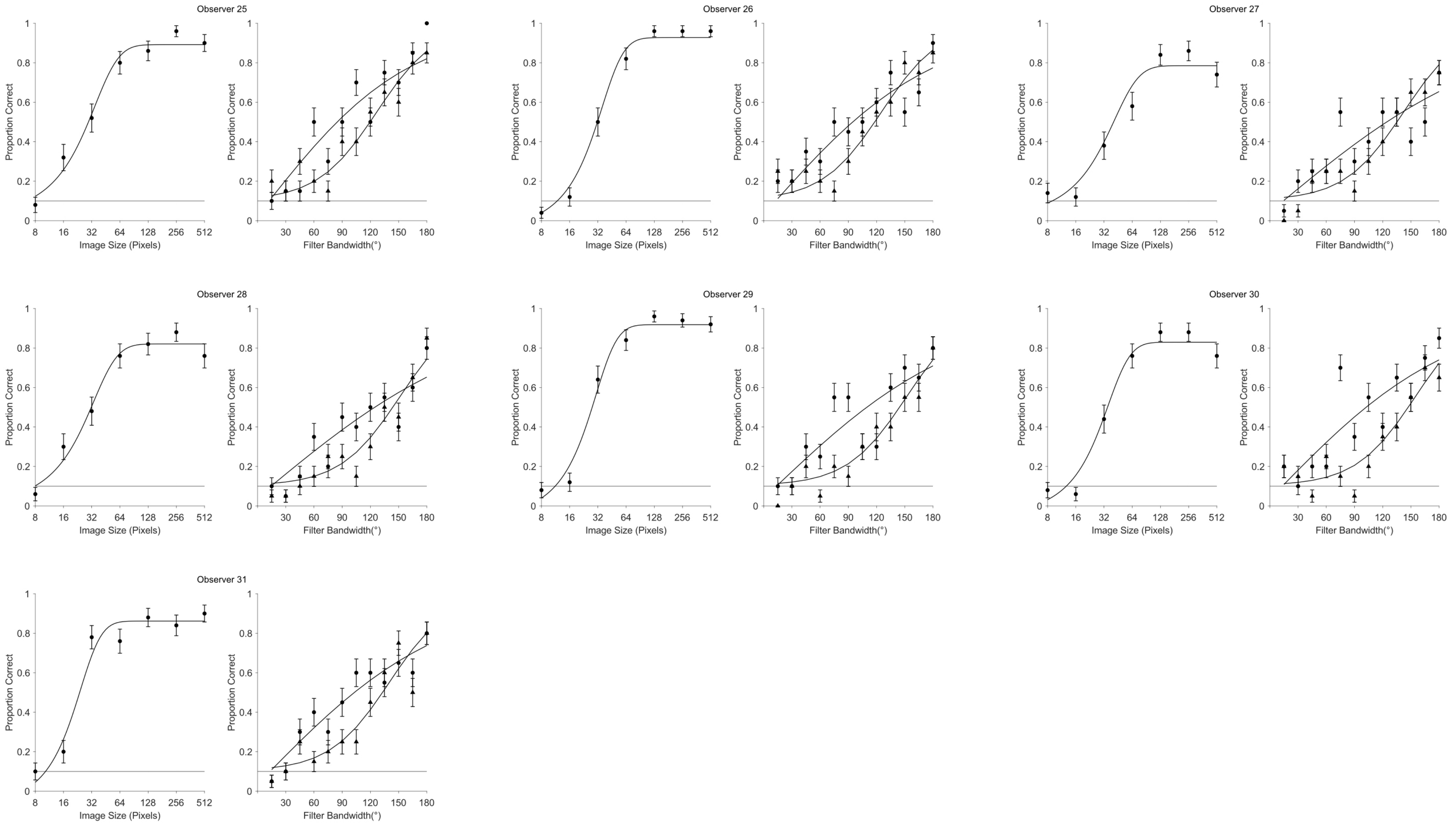
Observer 25
Observer 26
Observer 27
Observer 28
Observer 29
Observer 30
Observer 31
Proportion Correct
0
0.2
0.4
0.6
0.8
1
8
16
32
64
128
256
512
Image Size (Pixels)
30
60
90
120
150
180
Filter Bandwidth(°)

Caption for Supplementary Figure S2. Visualizations of Group by Filter Orientation differences. a. Box and whisker plots of horizontal selectivity for the average proportion correct obtained from 15°-90° filtered target stimuli. **b.** Plot of horizontal selectivity (difference in performance between horizontal and vertical filter orientations) for normal observers (solid line) and SRs (dashed line). Error bars represent the FDR-corrected 95% CI about the mean at each data point; the bold horizontal line above the groups' selectivity functions indicates bandwidths at which normal observers and SRs displayed significant horizontal selectivity.

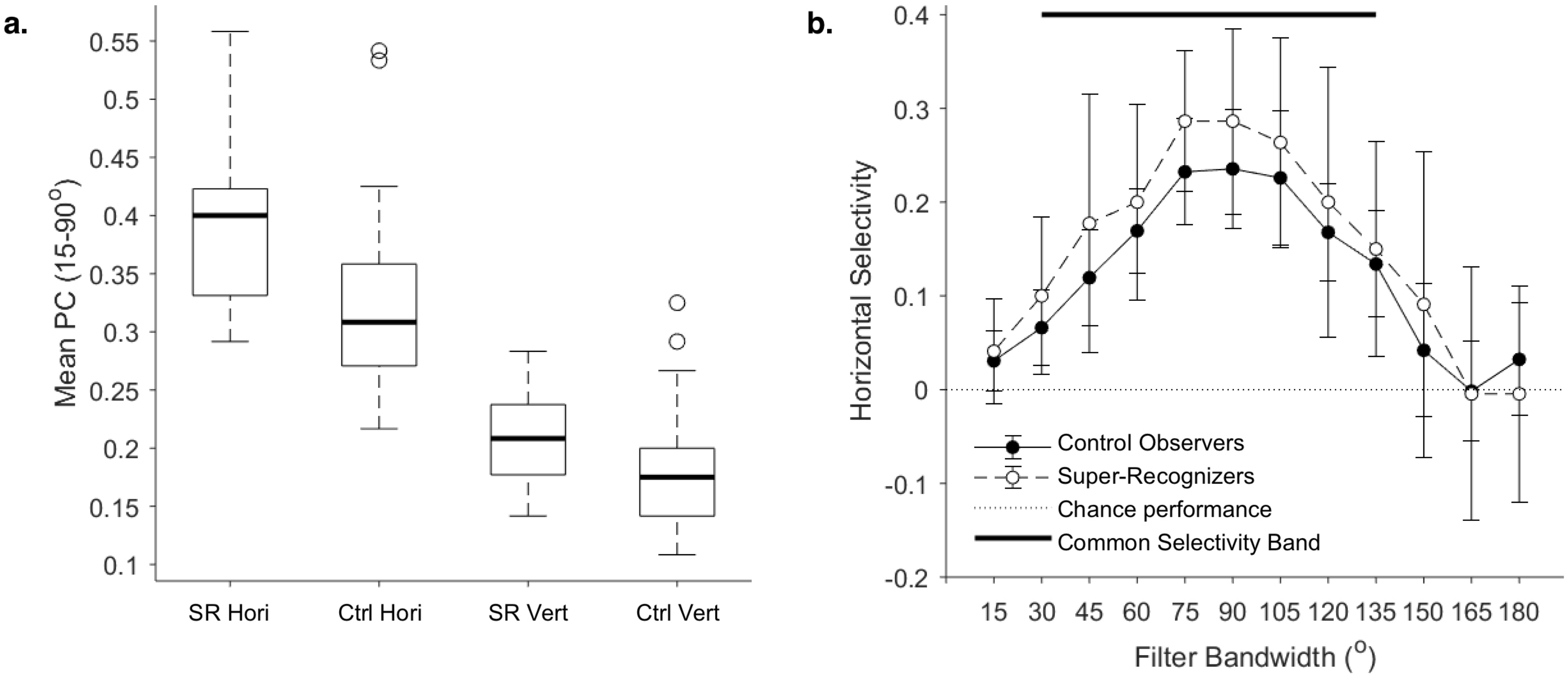
a.
Mean PC (15-90°)
0.55
0.5
0.45
0.4
0.35
0.3
0.25
0.2
0.15
0.1
SR Hori
Ctrl Hori
SR Vert
Ctrl Vert
b.
Horizontal Selectivity
0.4
0.3
0.2
0.1
0
-0.1
-0.2
Control Observers
Super-Recognizers
Chance performance
Common Selectivity Band
15
30
45
60
75
90
105
120
135
150
165
180
Filter Bandwidth (°)
